# Convergent evolution of the gut microbiome in marine carnivores

**DOI:** 10.1002/ece3.9373

**Published:** 2022-10-01

**Authors:** Xibao Wang, Xiaoyang Wu, Yongquan Shang, Xuesong Mei, Shengyang Zhou, Qinguo Wei, Guolei Sun, Yuehuan Dong, Honghai Zhang

**Affiliations:** ^1^ College of Life Sciences Qufu Normal University Qufu China

**Keywords:** convergent evolution, gut microbiome, marine carnivores, marine habitat

## Abstract

The gut microbiome can help the host adapt to a variety of environments and is affected by many factors. Marine carnivores have unique habitats in extreme environments. The question of whether marine habitats surpass phylogeny to drive the convergent evolution of the gut microbiome in marine carnivores remains unanswered. In the present study, we compared the gut microbiomes of 16 species from different habitats. Principal component analysis (PCA) and principal coordinate analysis (PCoA) separated three groups according to their gut microbiomes: marine carnivores, terrestrial carnivores, and terrestrial herbivores. The alpha diversity and niche breadth of the gut microbiome of marine carnivores were lower than those of the gut microbiome of terrestrial carnivores and terrestrial herbivores. The gut microbiome of marine carnivores harbored many marine microbiotas, including those belonging to the phyla Planctomycetes, Cyanobacteria, and Proteobacteria, and the genus *Peptoclostridium*. Collectively, these results revealed that marine habitats drive the convergent evolution of the gut microbiome of marine carnivores. This study provides a new perspective on the adaptive evolution of marine carnivores.

## Graphical abstract

To date, the question of whether marine habitats surpass phylogeny to drive the convergent evolution of the gut microbiome in marine carnivores has not been answered. Thus, in the present study, we investigated and compared the published gut microbiome data (16S rRNA) of four marine carnivores, five terrestrial carnivores, and seven terrestrial herbivores. Our findings proved our hypothesis that marine habitats drive the convergent evolution of the gut microbiome of marine mammals.

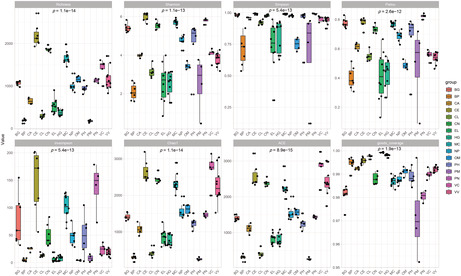

## 1 INTRODUCTION

Marine carnivores include species belonging to the orders Cetacea and Pinnipedia, genus *Enhydra*, and species *Ursus maritimus*, and they are united by lifestyle rather than evolutionary history (Erwin et al., 2017). They have undergone significant habitat transitions during their evolution (Williams, 1999), and the order Cetacea has dramatically changed from herbivorous to carnivorous (Wang et al., 2016). Furthermore, the marine environment is a unique habitat, as its temperature is lower and salinity is higher than those of the terrestrial environment (Liu et al., 2019). Therefore, marine carnivores are ideal models for investigating convergent evolution (Uhen, 2007). Many studies have focused on the adaptive evolution of marine carnivores using genomics (Noh et al., 2022; Yim et al., 2014), microbiomics (Dudek et al., 2022; Glaeser et al., 2022), and transcriptomics (Toren et al., 2020). Based on phylogenetic independent contrasts analysis, Wang, Shang, Wu, et al. (2022) found that the evolutionary rate of marine Cetartiodactyla mitochondrial protein‐coding genes was significantly higher than terrestrial Cetartiodactyla. Noh et al. (2022) found that *SUMO2* and *EP300* (hypoxia genes) were the most significant genes in the Weddell seal (*Leptonychotes weddellii*) compared to other placental mammals. The bottlenose dolphin (*Tursiops truncatus*) possesses a unique microbiome compared to that of other mammals and is similar to carnivorous marine fishes (Soverini et al., 2016). Based on comparative genomic analysis, Foote et al. (2015) discovered that convergent amino acid substitutions are widespread in the genome of marine carnivores, and a subset of positive selection evolutionary genes was putatively associated with marine phenotypes. Thus, the marine habitat drives the convergent evolution of marine mammal genes (including those of *Odobenus rosmarus*, *Tursiops truncates*, *Orcinus orca*, and *Trichechus manatus latirostris*).

The gut microbiome is an important factor for host adaptations to the environment (Wang et al., 2019; Wang, Shang, Wei, et al., 2022; Wang, Shang, Wu, et al., 2022; Wang, Wu, et al., 2022). Wang, Shang, Wu, et al. (2022) found that the gut microbiome function of red and corsac foxes can help hosts adapt to different environmental niches. Moreover, to adapt to plateau environments, short‐chain fatty acid (SCFA)‐producing bacteria are significantly enriched in the host gut (Li et al., 2016; Zhang et al., 2016). The gut microbiome also plays an important role in host health and survival (Davies et al., 2022; Gentile & Weir, 2018) and is dependent on various factors, such as diet (Greene et al., 2020; Wu et al., 2022), phylogeny (Sun et al., 2021; Wang et al., 2019), and habitat (Gacesa et al., 2022). Previous studies have shown that mammal gut microbiomes are strongly correlated with host phylogeny (Amato et al., 2019; Ley et al., 2008). In other words, mammals with closer phylogenetic relationships have similar gut microbiome compositions (Gregor et al., 2022). However, some influencing factors can surpass phylogeny to drive the convergent evolution of the mammalian gut microbiome (Huang et al., 2021; Song et al., 2020; Yao et al., 2021). For example, high altitude drives the convergent evolution of indicator microbiota in the gut microbiome of ungulates (Zhang et al., 2016). The gut microbiome was found to be similar among myrmecophagous species, although their phylogenetic relationships were distant (Delsuc et al., 2014). A bamboo diet was shown to drive gut microbiome convergence between the giant panda (*Ailuropoda melanoleuca*) and red panda (*Ailurus fulgens*) (Huang et al., 2021). Surprisingly, Proteobacteria were found to be the dominant phylum in bats and birds and were driven by flight behavior (Song et al., 2020).

Thus, extreme environments, special feeding habits, or behaviors can drive convergent evolution of the gut microbiome of species with the distant phylogenetic relationships. Under a broader phylogeny, it remains unclear whether marine habitats drive the convergent evolution of the gut microbiota of marine carnivores. Based on previous studies, we hypothesized that marine habitats drive the convergent evolution of the gut microbiome of marine carnivores. Therefore, we studied and compared the published gut microbiome (16S rRNA gene) data of four marine carnivores, five terrestrial carnivores, and seven terrestrial herbivores. Our findings helped explain these scientific problems and provide a new perspective for understanding the adaptation of marine carnivores to the marine environment.

## 2 MATERIALS AND METHODS

### 2.1 Species sampling and 16S rRNA gene sequence data

We analyzed the gut microbiomes of 108 samples representing 16 species belonging to nine families and 14 genera to explore the convergent evolution of the gut microbiome. 16S rRNA gene data of the gut microbiome of the nine species (*Cuon alpinus* [Wu et al., 2016], *Canis lupus* [Wu et al., 2017], *Vulpes Vulpes* [Wang, Shang, Wei, et al., 2022; Wang, Shang, Wu, et al., 2022; Wang, Wu, et al., 2022], *V. Corsac* [Wang, Shang, Wei, et al., 2022; Wang, Shang, Wu, et al., 2022; Wang, Wu, et al., 2022], *Cervus elaphus* [Wang et al., 2019], *Ovis musimon* [Sun et al., 2019], *Pantholops hodgsonii* [Wang, Shang, Wei, et al., 2022; Wang, Shang, Wu, et al., 2022; Wang, Wu, et al., 2022], *Pseudois nayaur* [Wang, Shang, Wei, et al., 2022; Wang, Shang, Wu, et al., 2022; Wang, Wu, et al., 2022], and *Bos grunniens* [Wang, Shang, Wei, et al., 2022; Wang, Shang, Wu, et al., 2022; Wang, Wu, et al., 2022]) were obtained by sequencing in our laboratory. Other 16S rRNA gene data (*C. Nippon* [Guan et al., 2017], *Moschus chrysogaster* [Sun et al., 2020], *Halichoerus grypus* [Watkins et al., 2022], *Nyctereutes procyonoides* [Ishida‐Kuroki et al., 2020], *Enhydra lutris nereis* [Dudek et al., 2022], *Balaenoptera physalus*, and *Physeter microcephalus* [Glaeser et al., 2022]) were downloaded from the NCBI SRA database (www.ncbi.nlm.nih.gov). The 16S rRNA gene sequences are listed in Appendix S1. Based on diet and habitat, these species were divided into three groups: terrestrial herbivores (TH group; *C. elaphus*, *O. musimon*, *P. hodgsonii*, *P. nayaur*, *B. grunniens*, *C. Nippon*, and *M. chrysogaster*), terrestrial carnivore (TC group; *C. alpinus*, *C. lupus*, *V. Vulpes*, *V. Corsac*, and *N. procyonoides*), and marine carnivore (MM group; *H. grypus*, *B. physalus*, *P. microcephalus*, and *E. lutris nereis*). Except for the data on *C. alpinus*, *C. lupus*, and *O. musimon*, all sample data were obtained from wild individuals. In previous studies, *C. alpinus*, *C. lupus*, and *O. musimon* were captive individuals (not treated with antibiotics) (Sun et al., 2021; Wu et al., 2016, 2017).

### 2.2 Sequence processing and statistical analyses

The paired‐end reads of the 16S rRNA gene was sequenced using a high‐throughput sequencing platform. The MOTHUR (Schloss et al., 2009) software was used to merge all 16S rRNA gene data. To avoid sequencing inaccuracy, the Parallel‐Meta Suite (PMS; V 3.7; Chen et al., 2022) was used to denoise (Callahan et al., 2017) and remove chimeras (Edgar et al., 2011). To eliminate the effect of using different sequencing intervals and sequencing depths of 16S rRNA data, PMS was used to cluster the sequences into operational taxonomic units (OTUs, with the conventional criterion of 97% sequence identity) and annotate the taxonomy (GreenGenes V13‐8) of each species, and the relative microbiome abundance table of each sample was obtained. This table was used as an intermediate result and reanalyzed by PMS to obtain OTU relative abundance tables and each taxon relative abundance table for all species. Based on the OTU level, alpha (α) diversity, principal component analysis (PCA), principal coordinate analysis (PCoA), gut microbiome niche breadth, and analysis of similarities (Anosim) were plotted using the Tutools platform (http://www.cloudtutu.com). Alpha diversity indexes were used to analyze the gut microbiome diversity between species. The gut microbiome niche breadth was used to judge whether a species is specialized. We used PCA, PCoA, and Anosim to verify whether the gut microbiome composition of species in different habitats was different. The Tutools platform was also used to perform the Kruskal‐Wallis test (*q* < .01; false discovery rate [FDR] method to correct decisions) to detect differences in the abundance of the gut microbiota between groups.

## 3 RESULTS

### 3.1 Overview of the 16S rRNA gene data

**FIGURE 1:**
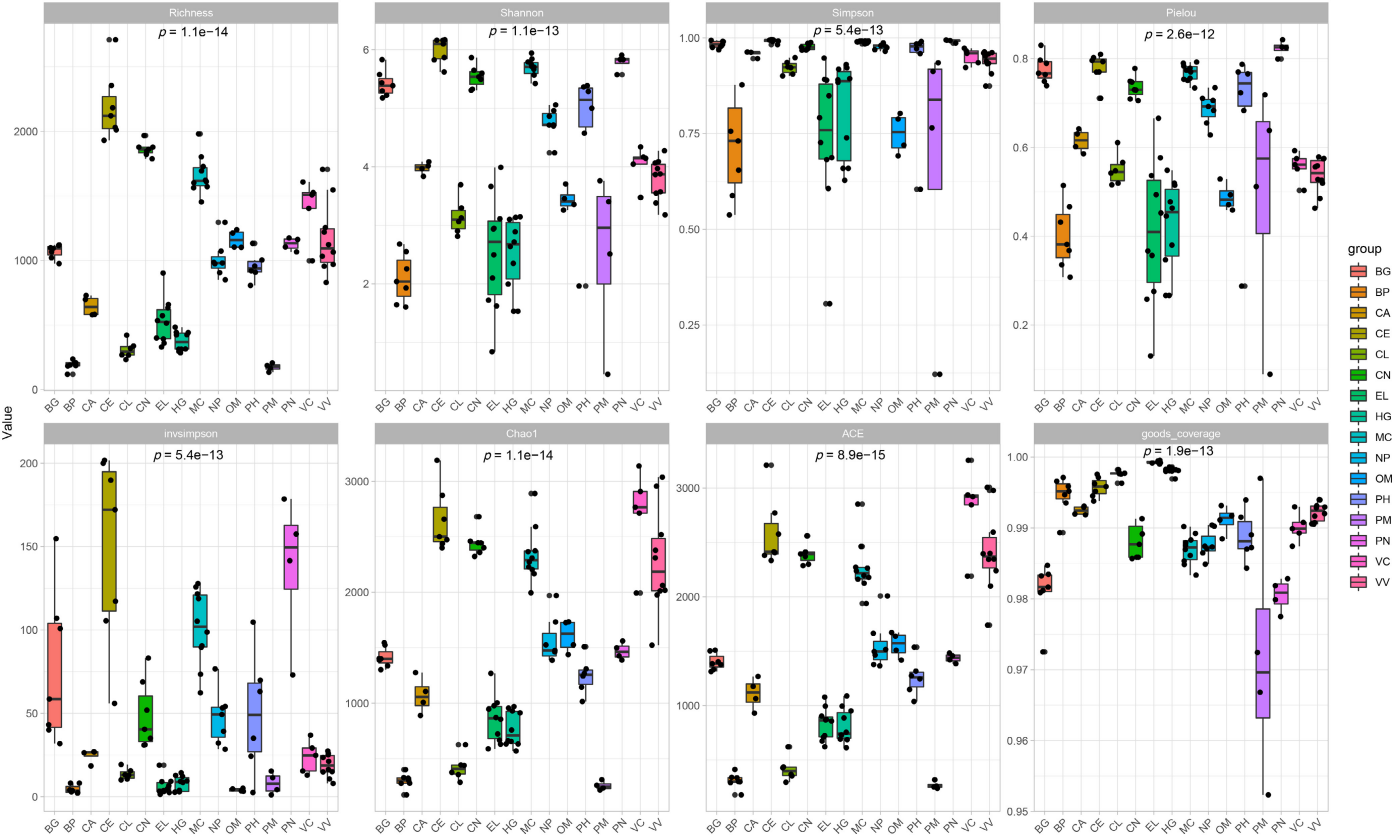
Kruskal‐Wallis test of gut microbiome alpha diversity between species. The abscissa is the species, and the ordinate is the numerical value. *p* Value less than .05 indicates that the difference between groups is significant.

After quality control, a total of 8,011,810 effective tags were obtained from 108 samples. Each sample contained an average of 74,183 tags. The good coverage index of all samples was more than 96.5% (Figure 1), which showed that the gut microbiomes were sufficient for subsequent analysis and also effectively represented those in the 16 species.

### 3.2 Alpha diversity and niche breadth of the gut microbiome

The alpha index (including Richness, Shannon, Simpson, Pielou, Invsimpson, Chao1, and ACE indices) boxplot between species showed that the alpha diversity of the gut microbiome of the investigated marine carnivores (*H. grypus*, HG; *B. physalus*, BP; *P. microcephalus*, PM; and E. *lutris nereis*, EL) was significantly (Kruskal–Wallis test, *p* < .01) lower than that of the investigated terrestrial carnivores (*C. alpinus*, CA; *C. lupus*, CL; *V. vulpes*, VV; *V. corsac*, VC; *N. procyonoides*, NP) and terrestrial herbivores (*C. elaphus*, CE; *O. musimon*, OM; *P. hodgsonii*, PH; *P. nayaur*, PN; *B. grunniens*, BG; *C. nippon*, CN; *M. chrysogaster*, MC). The alpha diversity of terrestrial herbivores was overall the highest, followed by that of terrestrial carnivores (Figure 1).

**FIGURE 2:**
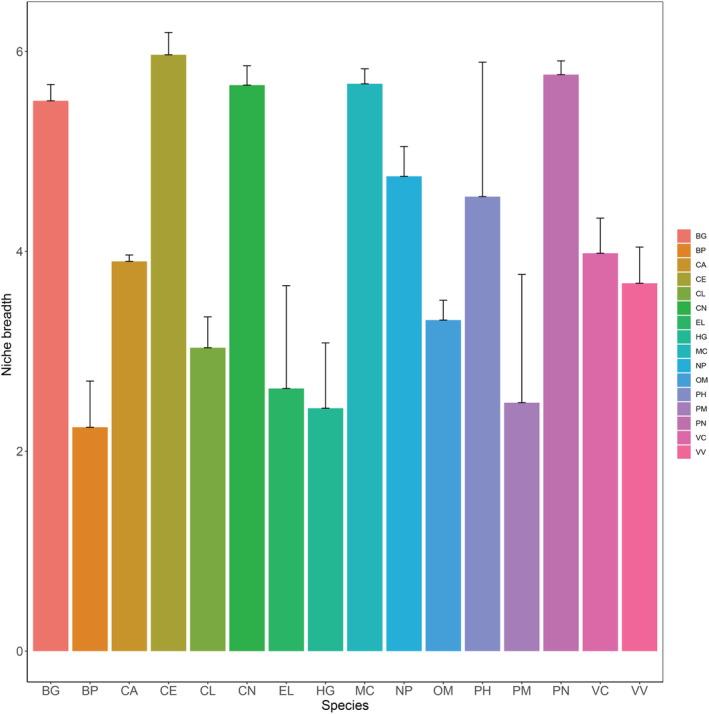
Niche breadth of the gut microbiome between species. The abscissa is the species, and the ordinate is the numerical value of niche breadth.

Furthermore, the degree of specialization of mammalian gut microbiomes in different habitats was characterized using the niche breadth of the gut microbiome. Overall, the niche breadth of the gut microbiome of marine carnivores was the lowest, and that of terrestrial herbivores was the highest (Figure 2). This result demonstrated that the gut microbiome of marine carnivores is specialized.

### 3.3 Cluster analyses

**FIGURE 3:**
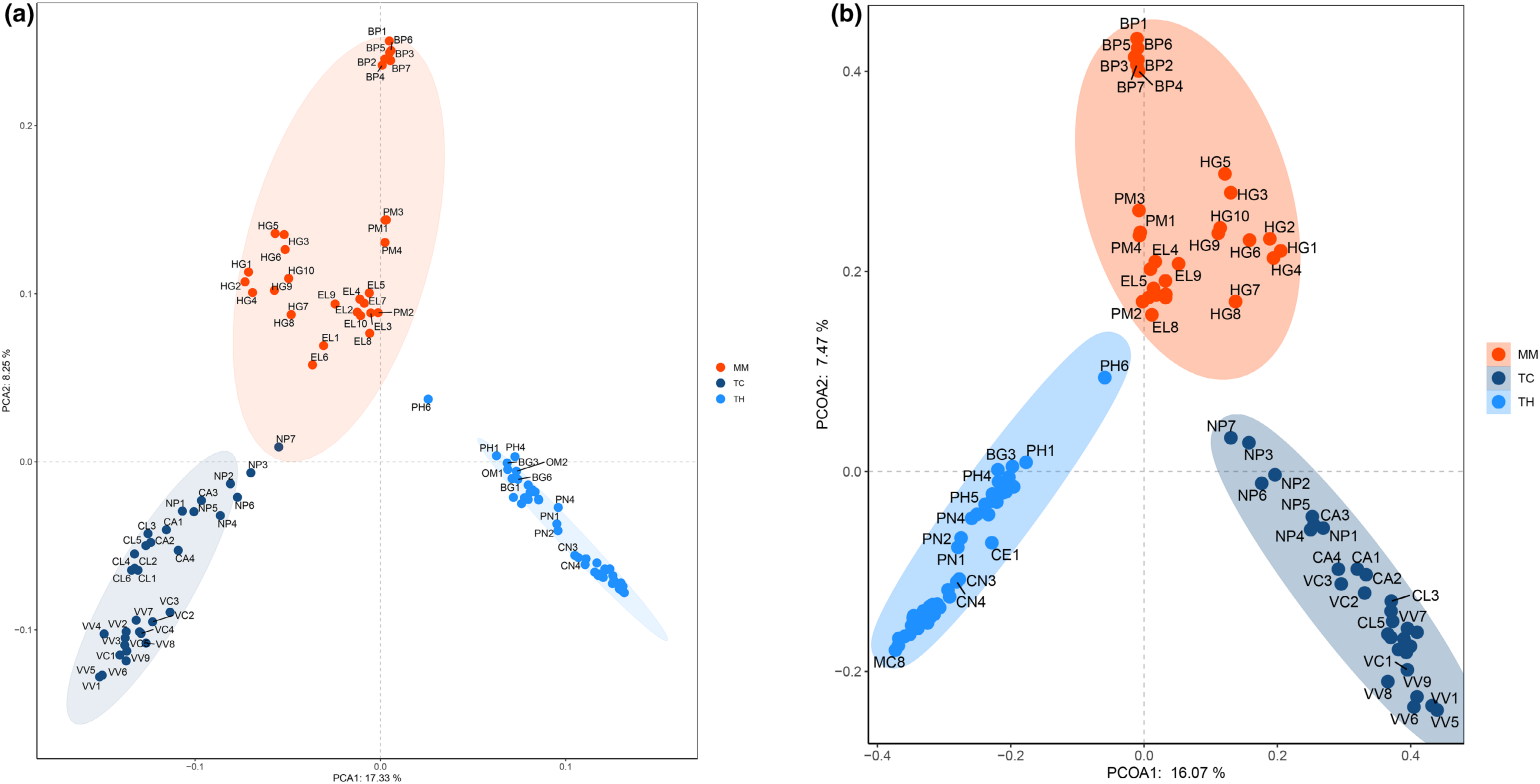
Principal component analysis (PCA; a) and principal coordinate analysis (PCoA; b) of gut microbiome composition. Each ellipse represents the gut microbiome of a group.

**FIGURE 4:**
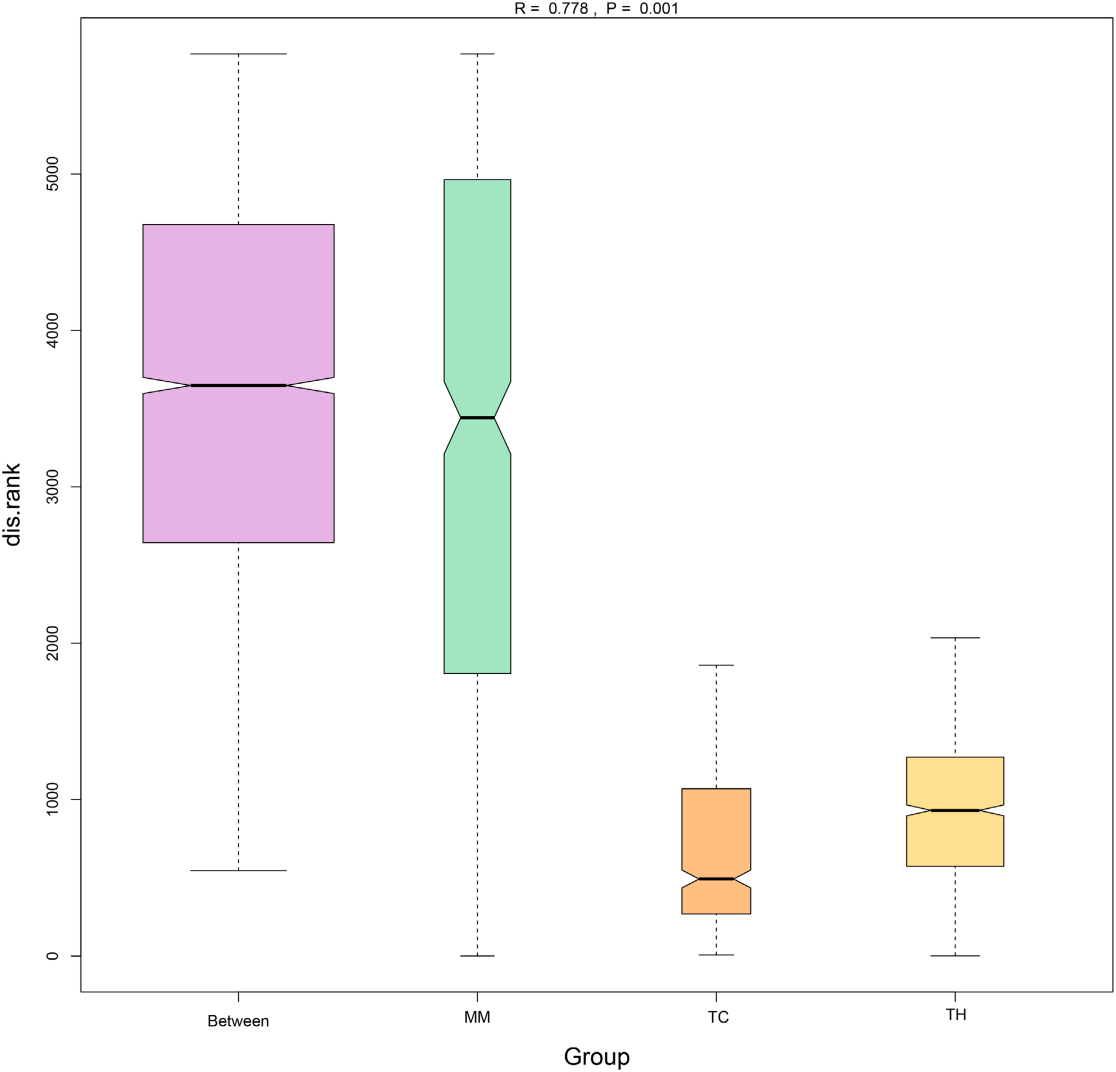
Analysis of similarities (Anosim) between groups. The abscissa is the groups, and the ordinate is the numerical value of distance rank. *R* value greater than zero indicates that the difference between groups is greater than that within groups. *p* value less than .05 indicates that the difference between the groups is significant.

Cluster analyses can be used to gather similar samples in a group. Therefore, we used cluster analyses to determine whether the marine environment drives the convergent evolution of the gut microbiome in marine carnivores. Based on the OTU level, the gut microbiome compositions of the MM, TC, and TH groups were separated in the PCA plot (Figure 3a). We used PCoA (Figure 3b) to verify this result. PCoA also showed that according to their gut microbiomes, the investigated animals could be divided into three groups: marine carnivores, terrestrial carnivores, and terrestrial herbivores. Anosim demonstrated that the gut microbiome compositions of the MM, TC, and TH groups were significantly different (*R* = .778, *p* = .001) according to Bray‐Curtis distances (Figure 4). These results indicated that marine habitats drive the convergent evolution of the gut microbiome in marine carnivores.

### 3.4 Gut microbiome composition

**FIGURE 5:**
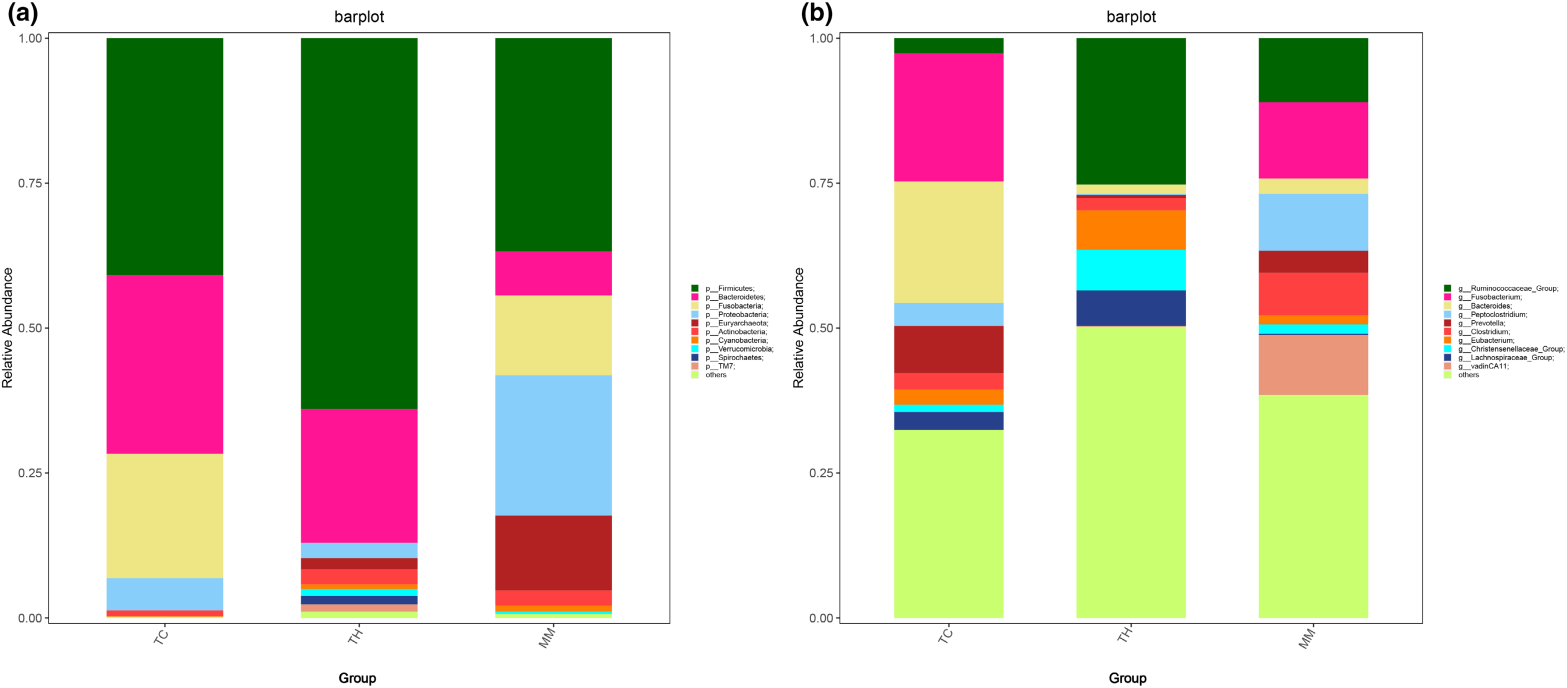
Gut microbiome composition between groups at the phylum (a) and genus (b) levels. Each bar represents the top 10 bacterial species sorted by relative abundance in each group.

At the phylum level, Firmicutes (MM, 36.72%; TC, 40.83%; TH, 60.92%) dominated the gut microbiome of the three groups. Bacteroidetes was the second most dominant phylum in the TC (30.87%) and TH groups (21.97%), and Proteobacteria was the second most dominant phylum in the MM group (24.18%). The third most dominant phylum in the TC (21.42%) and MM groups (13.75%) was Fusobacteria, while in the TH group (2.49%), it was Proteobacteria (Figure 5a). Notably, the relative abundance of the top three phyla accounted for 74.65%, 84.65%, and 85.38 of the bacterial community in the MM, TC, and TH groups, respectively. At the genus level, *Fusobacterium* was predominant in the MM (10.86%) and TC (21.43%) groups, while *Ruminococcaceae_Group* (24.25%) was the most abundant in the TH group (Figure 5b).

### 3.5 Discrepancies in the gut microbiome between groups

**FIGURE 6:**
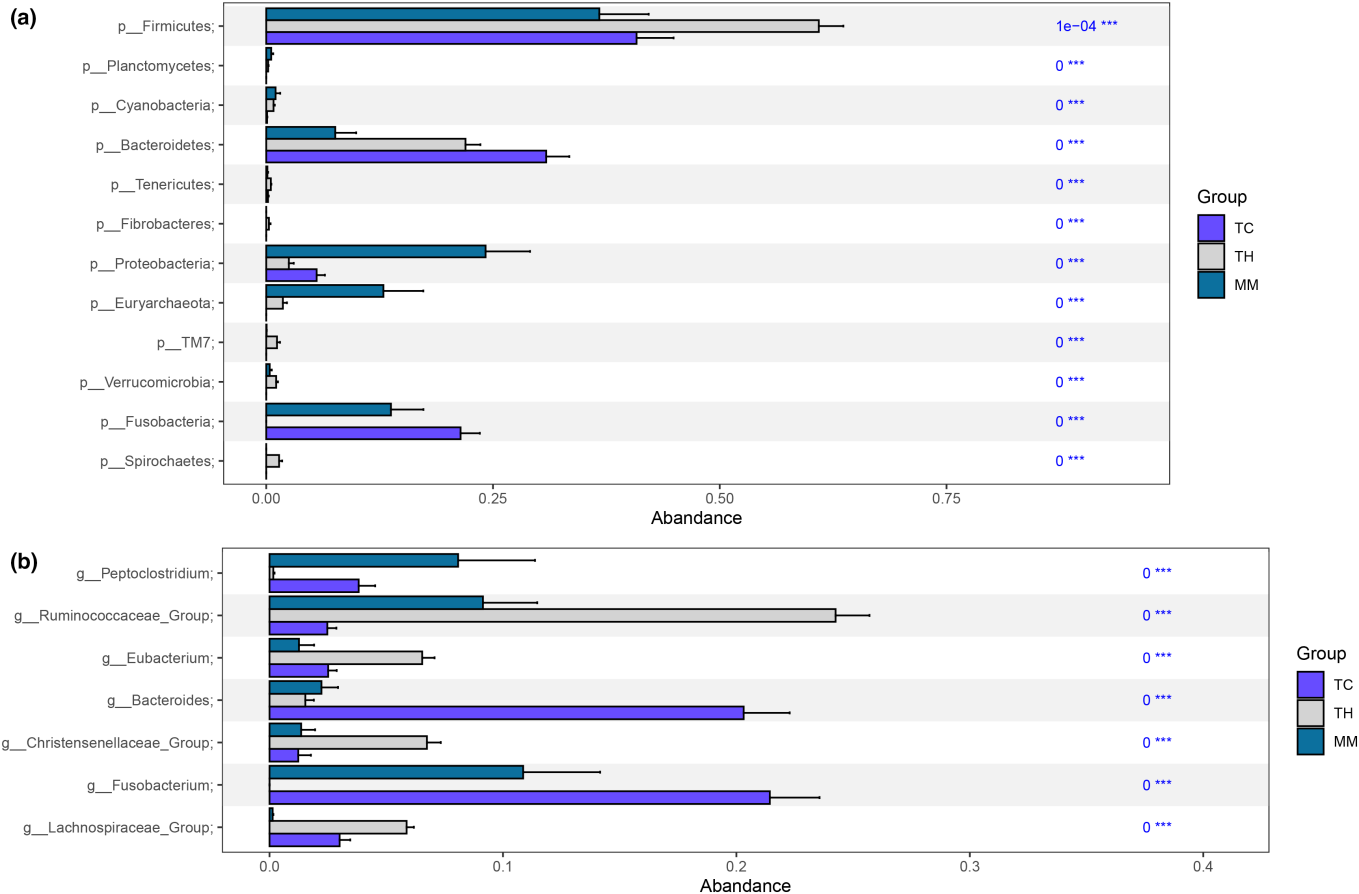
Kruskal‐Wallis test at the phylum (a) and genus (b) levels between groups. *p* value less than .05 indicates that the difference between the groups is significant (the numbers in the figure are *p* values; ***p < .001). Different colors represent different groups.

We used the Kruskal–Wallis test (*q* < .01) to characterize significantly enriched microbiota among the three groups. The phyla Firmicutes, Tenericutes, Fibrobacteres, TM7, Verrucomicrobia, and Spirochaetes were significantly enriched in the TH group; Planctomycetes, Cyanobacteria, Proteobacteria, and Euryarchaeota were significantly enriched in the MM group; and Bacteroidetes and Fusobacteria were significantly enriched in the TC group (*q* < .01) (Figure 6a). These phyla, except for Tenerictes and Planctomycetes, were the top 10 dominant phyla in the studied microbiomes.

At the genus level (Figure 6b), *Peptoclostridium* was significantly enriched in the MM group (*q* < .01); *Ruminococcaceae_Group*, *Eubacterium, Christensenellaceae_Group*, and *Lachnospiraceae_Group* were significantly enriched in the TH group; and *Bacteroides* and *Fusobacterium* were significantly enriched in the TC group (*q* < .01). These genera were among the top 10 genera with the highest relative abundances in the three groups. These results indicated that different habitats shaped the differences in the gut microbiome composition among the studied animals.

## 4 DISCUSSION

In the present study, we characterized 108 samples representing 16 species belonging to 8 families and 13 genera. A big dataset effectively eliminates the influence of abnormal individuals on the results. We obtained 8,011,810 effective tags, and the good coverage index was higher than 96.5% for all species. These results indicated a greater degree of coverage of the gut microbiome and also showed that the subsequent biometric analyses were reasonable. By comparing the gut microbiota across the three groups, we suggest that the different habitats affect the gut microbiome composition of the species. Especially, marine habitats can surpass phylogeny to drive the convergent evolution of gut microbiome composition in marine carnivores.

Our results showed that the alpha diversity (including Richness, Shannon, Simpson, Pielou, Invsimpson, Chao1, and ACE indices) of the MM group was significantly lower than those of the TH and TC groups. These results were consistent with those reported in previous studies (Bai et al., 2021; Nishida & Ochman, 2018). This could be related to the marine environment and host lifestyle (especially, land to the sea) (Thewissen et al., 2007). In addition to alpha diversity, the gut microbiome niche breadth of the MM group was lower than that of the TH and TC groups. Because of their habitat and evolutionary history, marine carnivores are highly specialized species (Hindle, 2020), and their specific gut microbiome can help them adapt to their unique habitats.

According to the PCA and PCoA, marine carnivores were clustered in the same group, whereas terrestrial carnivores and terrestrial herbivores gathered in a separate group. Although HG, EL, and terrestrial carnivores have close phylogenetic relationships, the gut microbiome compositions of HG and EL were similar to those of BP and PM. These results revealed that marine habitats could surpass phylogeny to drive the convergent evolution of the gut microbiome in marine carnivores.

Compared with those in the TC and TH groups, Planctomycetes, Proteobacteria, and Cyanobacteria were found to be significantly enriched in the gut microbiome of the MM group. Planctomycetes are mainly aquatic bacteria (Peeters et al., 2020) that widely exist in different marine environments (Shu & Jiao, 2008). Cyanobacteria play important roles as photosynthesis, nitrogen fixers, and producers of biologically active substances, and are more abundant in various marine ecosystems than in terrestrial ones (Andreeva et al., 2020; Sunagawa et al., 2015). Proteobacteria is the most important phylum in marine ecosystems (Sunagawa et al., 2015). Proteobacteria and Cyanobacteria were among the top 10 phyla in the MM group; Proteobacteria was the second most abundant phylum in this group. Furthermore, the genus *Peptoclostridium* was significantly enriched in the MM group compared with that in the TC group and has previously been isolated from marine sediments (Galperin et al., 2016). During the long process of evolution, marine microbiota may have colonized the gut of marine carnivores. In addition, the life history of marine carnivores increases the possibility of marine microbiota colonization. These bacteria may have played an important role in host adaptation to the marine environment.

## 5 CONCLUSION

In summary, marine carnivores have the same pattern of gut microbiome niche breadth, α diversity, and colonization of marine microorganisms in their gut microbiome. Although the phylogenetic relationships among *P. microcephalus*, *B. physalus*, and terrestrial herbivores are closer than those among *P. microcephalus*, *B. physalus*, *H. grypus*, and *E. lutris nereis*, the gut microbiomes of marine carnivores were grouped together. Therefore, marine habitats can surpass phylogeny to drive the convergent evolution of the gut microbiome in marine carnivores. This study provides a new perspective on the adaptive evolution of marine carnivores.

## AUTHOR CONTRIBUTIONS

**Xibao Wang:** Data curation (equal); formal analysis (equal); project administration (equal); writing – original draft (equal); writing – review and editing (equal). **Xiaoyang Wu:** Formal analysis (equal). **Yongquan Shang:** Formal analysis (equal). **Xuesong Mei:** Formal analysis (supporting). **Shengyang Zhou:** Project administration (supporting). **Qinguo Wei:** Project administration (supporting). **Guolei Sun:** Formal analysis (supporting). **Yuehuan Dong:** Formal analysis (supporting). **Honghai Zhang:** Funding acquisition (lead); project administration (equal).

## CONFLICT OF INTEREST

No potential conflict of interest was reported by the authors.

## Supporting information


Appendix S1

## Data Availability

All 16S rRNA gene sequencing data used in this study were accessed through the SRA database using the accession numbers and DOI numbers in Appendix S1.

## References

[ece39373-bib-0001] Amato, K. R. , Sanders, G. J. , Song, S. J. , Nute, M. , Metcalf, J. L. , Thompson, L. R. , Morton, J. T. , Amir, A. , McKenzie, V. J. , Humphrey, G. , Gogul, G. , Gaffney, J. , Baden, A. L. , Britton, G. A. O. , Cuozzo, F. P. , Fiore, A. D. , Dominy, N. J. , Goldberg, T. L. , Gomez, A. , … Leigh, S. R. (2019). Evolutionary trends in host physiology outweigh dietary niche in structuring primate gut microbiomes. The ISME Journal, 13(3), 576–587. 10.1038/s41396-018-0175-0 29995839 PMC6461848

[ece39373-bib-0002] Andreeva, N. A. , Melnikov, V. V. , & Snarskaya, D. D. (2020). The role of cyanobacteria in marine ecosystems. Russian Journal of Marine Biology, 46(3), 154–165. 10.1134/S1063074020030025

[ece39373-bib-0003] Bai, S. , Zhang, P. , Zhang, C. , Du, J. , Du, X. , Zhu, C. , Liu, J. , Xie, P. , & Li, S. (2021). Comparative study of the gut microbiota among four different marine mammals in an aquarium. Frontiers in Microbiology, 12, 769012. 10.3389/fmicb.2021.769012 34745077 PMC8567075

[ece39373-bib-0004] Callahan, B. J. , McMurdie, P. J. , & Holmes, S. P. (2017). Exact sequence variants should replace operational taxonomic units in marker‐gene data analysis. The ISME Journal, 11(12), 2639–2643. 10.1038/ismej.2017.119 28731476 PMC5702726

[ece39373-bib-0005] Chen, Y. , Li, J. , Zhang, Y. , Zhang, M. , Sun, Z. , Jing, G. , Huang, S. , & Su, X. (2022). Parallel‐meta suite: Interactive and rapid microbiome data analysis on multiple platforms. iMeta, 1(1), e1. 10.1002/imt2.1 PMC10989749 38867729

[ece39373-bib-0006] Davies, C. S. , Worsley, S. F. , Maher, K. H. , Komdeur, J. , Burke, T. , Dugdale, H. L. , & Richardson, D. S. J. M. (2022). Immunogenetic variation shapes the gut microbiome in a natural vertebrate population. Microbiome, 10(1), 1–22. 10.1186/s40168-022-01233-y 35256003 PMC8903650

[ece39373-bib-0007] Delsuc, F. , Metcalf, J. L. , Wegener Parfrey, L. , Song, S. J. , González, A. , & Knight, R. (2014). Convergence of gut microbiomes in myrmecophagous mammals. Molecular Ecology, 23(6), 1301–1317. 10.1111/mec.12501 24118574

[ece39373-bib-0008] Dudek, N. K. , Switzer, A. D. , Costello, E. K. , Murray, M. J. , Tomoleoni, J. A. , Staedler, M. M. , Tinker, M. T. , & Relman, D. A. (2022). Characterizing the oral and distal gut microbiota of the threatened southern sea otter (*Enhydra lutris nereis*) to enhance conservation practice. Conservation Science and Practice, 4(4), e12640. 10.1111/csp2.12640 35382031 PMC8979051

[ece39373-bib-0009] Edgar, R. C. , Haas, B. J. , Clemente, J. C. , Quince, C. , & Knight, R. (2011). UCHIME improves sensitivity and speed of chimera detection. Bioinformatics, 27(16), 2194–2200. 10.1093/bioinformatics/btr381 21700674 PMC3150044

[ece39373-bib-0010] Erwin, P. M. , Rhodes, R. G. , Kiser, K. B. , Keenan‐Bateman, T. F. , McLellan, W. A. , & Pabst, D. A. (2017). High diversity and unique composition of gut microbiomes in pygmy (*Kogia breviceps*) and dwarf (*K. sima*) sperm whales. Scientific Reports, 7(1), 7205. 10.1038/s41598-017-07425-z 28775301 PMC5543158

[ece39373-bib-0011] Foote, A. , Liu, Y. , Thomas, G. , Vinař, T. , Alföldi, J. , Deng, J. , Dugan, S. , van Elk, C. E. , Hunter, M. E. , Joshi, V. , Khan, Z. , Kovar, C. , Lee, S. L. , Lindblad‐Toh, K. , Mancia, A. , Nielsen, R. , Qin, X. , Qu, J. , Raney, B. J. , … Gibbs, R. A. (2015). Convergent evolution of the genomes of marine mammals. Nature Genetics, 47, 272–275. 10.1038/ng.3198 25621460 PMC4644735

[ece39373-bib-0012] Gacesa, R. , Kurilshikov, A. , Vich Vila, A. , Sinha, T. , Klaassen, M. , Bolte, L. , Andreu‐Sánchez, S. , Chen, L. , Collij, V. , Hu, S. , Dekens, J. A. M. , Lenters, V. C. , Björk, J. R. , Swarte, J. C. , Swertz, M. A. , Jansen, B. H. , Gelderloos‐Arends, J. , Jankipersadsing, S. , Hofker, M. , … Weersma, R. K. (2022). Environmental factors shaping the gut microbiome in a Dutch population. Nature, 604, 732–739. 10.1038/s41586-022-04567-7 35418674

[ece39373-bib-0013] Galperin, M. Y. , Brover, V. , Tolstoy, I. , & Yutin, N. (2016). Phylogenomic analysis of the family Peptostreptococcaceae (Clostridium cluster XI) and proposal for reclassification of Clostridium litorale (Fendrich et al. 1991) and Eubacterium acidaminophilum (Zindel et al. 1989) as Peptoclostridium litorale gen. nov. comb. nov. and Peptoclostridium acidaminophilum comb. nov. International Journal of Systematic and Evolutionary Microbiology, 66(12), 5506–5513. 10.1099/ijsem.0.001548 27902180 PMC5244501

[ece39373-bib-0014] Gentile, C. L. , & Weir, T. L. (2018). The gut microbiota at the intersection of diet and human health. Science, 362(6416), 776–780. 10.1126/science.aau5812 30442802 PMC13264711

[ece39373-bib-0015] Glaeser, S. P. , Silva, L. M. R. , Prieto, R. , Silva, M. A. , Franco, A. , Kämpfer, P. , Hermosilla, C. , Taubert, A. , & Eisenberg, T. (2022). A preliminary comparison on faecal microbiomes of free‐ranging large Baleen (*Balaenoptera musculus*, *B. physalus*, *B. borealis*) and toothed (*Physeter macrocephalus*) whales. Microbial Ecology, 83(1), 18–33. 10.1007/s00248-021-01729-4 33745062 PMC8881428

[ece39373-bib-0016] Greene, L. K. , Williams, C. V. , Junge, R. E. , Mahefarisoa, K. L. , Rajaonarivelo, T. , Rakotondrainibe, H. , O'Connell, T. M. , & Drea, C. M. (2020). A role for gut microbiota in host niche differentiation. The ISME Journal, 14(7), 1675–1687. 10.1038/s41396-020-0640-4 32238913 PMC7305313

[ece39373-bib-0017] Gregor, R. , Probst, M. , Eyal, S. , Aksenov, A. , Sasson, G. , Horovitz, I. , Dorrestein, P. C. , Meijler, M. M. , & Mizrahi, I. (2022). Mammalian gut metabolomes mirror microbiome composition and host phylogeny. The ISME Journal, 16(5), 1262–1274. 10.1038/s41396-021-01152-0 34903850 PMC9038745

[ece39373-bib-0018] Guan, Y. , Yang, H. , Han, S. , Feng, L. , Wang, T. , & Ge, J. (2017). Comparison of the gut microbiota composition between wild and captive sika deer (*Cervus nippon hortulorum*) from feces by high‐throughput sequencing. AMB Express, 8(1), 15. 10.1186/s13568-017-0517-8 PMC5799092 29404873

[ece39373-bib-0019] Hindle, A. G. (2020). Diving deep: Understanding the genetic components of hypoxia tolerance in marine mammals. Journal of Applied Physiology, 128(5), 1439–1446. 10.1152/japplphysiol.00846.2019 32324472

[ece39373-bib-0020] Huang, G. , Wang, X. , Hu, Y. , Wu, Q. , Nie, Y. , Dong, J. , Ding, Y. , Yan, L. , & Wei, F. (2021). Diet drives convergent evolution of gut microbiomes in bamboo‐eating species. Science China. Life Sciences, 64(1), 88–95. 10.1007/s11427-020-1750-7 32617829

[ece39373-bib-0021] Ishida‐Kuroki, K. , Takeshita, N. , Nitta, Y. , Chuma, T. , Maeda, K. , Shimoda, H. , Takano, A. , & Sekizaki, T. (2020). 16S rRNA gene amplicon sequence data from feces of five species of wild animals in Japan. Microbiology Resource Announcements, 9(22), e00368‐20. 10.1128/MRA.00368-20 32467273 PMC7256260

[ece39373-bib-0022] Ley, R. E. , Hamady, M. , Lozupone, C. , Turnbaugh, P. J. , Ramey, R. R. , Bircher, J. S. , Schlegel, M. L. , Tucker, T. A. , Schrenzel, M. D. , Knight, R. , & Gordon, J. I. (2008). Evolution of mammals and their gut microbes. Science, 320(5883), 1647–1651. 10.1126/science.1155725 18497261 PMC2649005

[ece39373-bib-0023] Li, K. , Dan, Z. , Gesang, L. , Wang, H. , Zhou, Y. , Du, Y. , Ren, Y. , Shi, Y. , & Nie, Y. (2016). Comparative analysis of gut microbiota of native Tibetan and Han populations living at different altitudes. PLoS One, 11(5), e0155863. 10.1371/journal.pone.0155863 27232599 PMC4883765

[ece39373-bib-0024] Liu, D. , Huang, J. , Wu, C. , Liu, C. , Huang, R. , Wang, W. , Yin, T. , Yan, X. , He, H. , & Chen, L. (2019). Purification, characterization, and application for preparation of antioxidant peptides of extracellular protease from Pseudoalteromonas sp. H2. Molecules, 24(18), 3373. 10.3390/molecules24183373 PMC6766936 31527535

[ece39373-bib-0025] Nishida, A. H. , & Ochman, H. (2018). Rates of gut microbiome divergence in mammals. Molecular Ecology, 27(8), 1884–1897. 10.1111/mec.14473 29290090 PMC5935551

[ece39373-bib-0026] Noh, H. J. , Turner‐Maier, J. , Schulberg, S. A. , Fitzgerald, M. L. , Johnson, J. , Allen, K. N. , Hückstädt, L. A. , Batten, A. J. , Alfoldi, J. , Costa, D. P. , Karlsson, E. K. , Zapol, W. M. , Buys, E. S. , Lindblad‐Toh, K. , & Hindle, A. G. (2022). The Antarctic Weddell seal genome reveals evidence of selection on cardiovascular phenotype and lipid handling. Communications Biology, 5(1), 140. 10.1038/s42003-022-03089-2 35177770 PMC8854659

[ece39373-bib-0027] Peeters, S. H. , Wiegand, S. , Kallscheuer, N. , Jogler, M. , Heuer, A. , Jetten, M. S. , Rast, P. , Boedeker, C. , Rohde, M. , & Jogler, C. (2020). Three marine strains constitute the novel genus and species Crateriforma conspicua in the phylum planctomycetes. Antonie Van Leeuwenhoek, 113(12), 1797–1809. 10.1007/s10482-019-01375-4 31894495

[ece39373-bib-0028] Schloss, P. D. , Westcott, S. L. , Ryabin, T. , Hall, J. R. , Hartmann, M. , Hollister, E. B. , Lesniewski, R. A. , Oakley, B. B. , Parks, D. H. , Robinson, C. J. , Sahl, J. W. , Stres, B. , Thallinger, G. G. , Van Horn, D. J. , & Weber, C. F. (2009). Introducing mothur: Open‐source, platform‐independent, community‐supported software for describing and comparing microbial communities. Applied and Environmental Microbiology, 75(23), 7537–7541. 10.1128/AEM.01541-09 19801464 PMC2786419

[ece39373-bib-0029] Shu, Q. , & Jiao, N. (2008). Different planctomycetes diversity patterns in latitudinal surface seawater of the open sea and in sediment. Russian Journal of Marine Biology, 46(2), 154–159. 10.1007/s12275-008-0002-9 18545964

[ece39373-bib-0030] Song, S. J. , Sanders, J. G. , Delsuc, F. , Metcalf, J. , Amato, K. , Taylor, M. W. , Mazel, F. , Lutz, H. L. , Winker, K. , Graves, G. R. , Humphrey, G. , Gilbert, J. A. , Hackett, S. J. , White, K. P. , Skeen, H. R. , Kurtis, S. M. , Withrow, J. , Braile, T. , Miller, M. , … Knight, R. (2020). Comparative analyses of vertebrate gut microbiomes reveal convergence between birds and bats. mBio, 11(1), e02901–e02919. 10.1128/mBio.02901-19 PMC6946802 31911491

[ece39373-bib-0031] Soverini, M. , Quercia, S. , Biancani, B. , Furlati, S. , Turroni, S. , Biagi, E. , Consolandi, C. , Peano, C. , Severgnini, M. , Rampelli, S. , Brigidi, P. , & Candela, M. (2016). The bottlenose dolphin (*Tursiops truncatus*) faecal microbiota. FEMS Microbiology Ecology, 92(4), fiw055. 10.1093/femsec/fiw055 26960390

[ece39373-bib-0032] Sun, G. , Xia, T. , Wei, Q. , Dong, Y. , Zhao, C. , Yang, X. , Zhang, L. , Wang, X. , Sha, W. , & Zhang, H. (2021). Analysis of gut microbiota in three species belonging to different genera (*Hemitragus*, *Pseudois*, and *Ovis*) from the subfamily Caprinae in the absence of environmental variance. Ecology and Evolution, 11(17), 12129–12140. 10.1002/ece3.7976 34522365 PMC8427585

[ece39373-bib-0033] Sun, G. , Zhang, H. , Wei, Q. , Zhao, C. , Yang, X. , Wu, X. , Xia, T. , Liu, G. , Zhang, L. , Gao, Y. , Sha, W. , & Li, Y. (2019). Comparative analyses of fecal microbiota in European mouflon (*Ovis orientalis musimon*) and blue sheep (*Pseudois nayaur*) living at low or high altitudes. Frontiers in Microbiology, 10, 1735. 10.3389/fmicb.2019.01735 31417526 PMC6682669

[ece39373-bib-0034] Sun, Y. , Sun, Y. , Shi, Z. , Liu, Z. , Zhao, C. , Lu, T. , Gao, H. , Zhu, F. , Chen, R. , Zhang, J. , Pan, R. , Li, B. , Teng, L. , & Guo, S. (2020). Gut microbiota of wild and captive alpine musk deer (*Moschus chrysogaster*). Frontiers in Microbiology, 10, 3156. 10.3389/fmicb.2019.03156 32038587 PMC6985557

[ece39373-bib-0035] Sunagawa, S. , Coelho, L. P. , Chaffron, S. , Kultima, J. R. , Labadie, K. , Salazar, G. , Djahanschiri, B. , Zeller, G. , Mende, D. R. , Alberti, A. , Cornejo‐Castillo, F. M. , Costea, P. I. , Cruaud, C. , di'Ovidio, F. , Engelen, S. , Ferrera, I. , Gasol, J. M. , Guidi, L. , Hildebrand, F. , … Bork, P. (2015). Structure and function of the global ocean microbiome. Science, 348(6237), 1261359. 10.1126/science.1261359 25999513

[ece39373-bib-0036] Thewissen, J. G. , Cooper, L. N. , Clementz, M. T. , Bajpai, S. , & Tiwari, B. N. (2007). Whales originated from aquatic artiodactyls in the Eocene epoch of India. Nature, 450(7173), 1190–1194. 10.1038/nature06343 18097400

[ece39373-bib-0037] Toren, D. , Kulaga, A. , Jethva, M. , Rubin, E. , Snezhkina, A. V. , Kudryavtseva, A. V. , Nowicki, D. , Tacutu, R. , Moskalev, A. A. , & Fraifeld, V. E. (2020). Gray whale transcriptome reveals longevity adaptations associated with DNA repair and ubiquitination. Aging Cell, 19(7), e13158. 10.1111/acel.13158 32515539 PMC7433004

[ece39373-bib-0038] Uhen, M. D. (2007). Evolution of marine mammals: Back to the sea after 300 million years. Advances in Integrative Anatomy and Evolutionary Biology, 290(6), 514–522. 10.1002/ar.20545 17516441

[ece39373-bib-0039] Wang, X. , Chen, Y. , Shang, Y. , Wu, X. , Wei, Q. , Chen, J. , Yan, J. , & Zhang, H. (2019). Comparison of the gut microbiome in red deer (*Cervus elaphus*) and fallow deer (*Dama dama*) by high‐throughput sequencing of the V3–V4 region of the 16S rRNA gene. ScienceAsia, 45(6), 515–524. 10.2306/scienceasia1513-1874.2019.45.515

[ece39373-bib-0040] Wang, X. , Shang, Y. , Wei, Q. , Wu, X. , Dou, H. , Zhang, H. , Zhou, S. , Sha, W. , Sun, G. , Ma, S. , & Zhang, H. (2022). Comparative analyses of the gut microbiome of two fox species, the red fox (*Vulpes Vulpes*) and Corsac fox (*Vulpes Corsac*), that occupy different ecological niches. Microbial Ecology, 83(3), 753–765. 10.1007/s00248-021-01806-8 34189610

[ece39373-bib-0041] Wang, X. , Shang, Y. , Wu, X. , Wei, Q. , Zhou, S. , Sun, G. , Mei, X. , Dong, Y. , Sha, W. , & Zhang, H. (2022). Divergent evolution of mitogenomics in Cetartiodactyla niche adaptation. Organisms Diversity & Evolution. 10.1007/s13127-022-00574-8

[ece39373-bib-0042] Wang, X. , Wu, X. , Shang, Y. , Gao, Y. , Li, Y. , Wei, Q. , Dong, Y. , Mei, X. , Zhou, S. , Sun, G. , Liu, L. , Lige, B. , Zhang, Z. , & Zhang, H. (2022). High‐altitude drives the convergent evolution of alpha diversity and indicator microbiota in the gut microbiomes of ungulates. Frontiers in Microbiology, 13, 953234. 10.3389/fmicb.2022.953234 35875556 PMC9301279

[ece39373-bib-0043] Wang, Z. , Xu, S. , Du, K. , Huang, F. , Chen, Z. , Zhou, K. , Ren, W. , & Yang, G. (2016). Evolution of digestive enzymes and RNASE1 provides insights into dietary switch of cetaceans. Molecular Biology and Evolution, 33(12), 3144–3157. 10.1093/molbev/msw191 27651393 PMC5100049

[ece39373-bib-0044] Watkins, C. A. , Gaines, T. , Strathdee, F. , Baily, J. L. , Watson, E. , Hall, A. J. , Free, A. , & Dagleish, M. P. (2022). A comparative study of the fecal microbiota of gray seal pups and yearlings‐a marine mammal sentinel species. Microbiology, 11(3), e1281. 10.1002/mbo3.1281 PMC9126079 35765184

[ece39373-bib-0045] Williams, T. M. (1999). The evolution of cost efficient swimming in marine mammals: Limits to energetic optimization. Philosophical Transactions of the Royal Society B: Biological Sciences, 354(1380), 193–201. 10.1098/rstb.1999.0371

[ece39373-bib-0046] Wu, X. , Wei, Q. , Wang, X. , Shang, Y. , & Zhang, H. (2022). Evolutionary and dietary relationships of wild mammals based on the gut microbiome. Gene, 808, 145999. 10.1016/j.gene.2021.145999 34627942

[ece39373-bib-0047] Wu, X. , Zhang, H. , Chen, J. , Shang, S. , Wei, Q. , Yan, J. , & Tu, X. (2016). Comparison of the fecal microbiota of dholes high‐throughput Illumina sequencing of the V3–V4 region of the 16S rRNA gene. Applied Microbiology and Biotechnology, 100(8), 3577–3586. 10.1007/s00253-015-7257-y 26728019

[ece39373-bib-0048] Wu, X. , Zhang, H. , Chen, J. , Shang, S. , Yan, J. , Chen, Y. , Tang, X. , & Zhang, H. (2017). Analysis and comparison of the wolf microbiome under different environmental factors using three different data of Next Generation Sequencing. Scientific Reports, 7(1), 11332. 10.1038/s41598-017-11770-4 28900198 PMC5596057

[ece39373-bib-0049] Yao, R. , Dai, Q. , Wu, T. , Yang, Z. , Chen, H. , Liu, G. , Zhu, Y. , Qi, D. , Yang, X. , Luo, W. , Gu, X. , Yang, X. , & Zhu, L. (2021). Fly‐over phylogeny across invertebrate to vertebrate: The giant panda and insects share a highly similar gut microbiota. Computational and Structural Biotechnology Journal, 19, 4676–4683. 10.1016/j.csbj.2021.08.025 34504662 PMC8390952

[ece39373-bib-0050] Yim, H.‐S. , Cho, Y. S. , Guang, X. , Kang, S. G. , Jeong, J.‐Y. , Cha, S.‐S. , Oh, H. M. , Lee, J. H. , Yang, E. C. , Kwon, K. K. , Kim, Y. J. , Kim, T. W. , Kim, W. , Jeon, J. H. , Kim, S. J. , Choi, D. H. , Jho, S. , Kim, H. M. , Ko, J. , … Lee, J. H. (2014). Minke whale genome and aquatic adaptation in cetaceans. Nature Genetics, 46(1), 88–92. 10.1038/ng.2835 24270359 PMC4079537

[ece39373-bib-0051] Zhang, Z. , Xu, D. , Wang, L. , Hao, J. , Wang, J. , Zhou, X. , Wang, W. , Qiu, Q. , Huang, X. , Zhou, J. , Long, R. , Zhao, F. , & Shi, P. (2016). Convergent evolution of rumen microbiomes in high‐altitude mammals. Current Biology, 26(14), 1873–1879. 10.1016/j.cub.2016.05.012 27321997

